# Brazilian forensic nursing: convergence of goals for the specialty’s next decade

**DOI:** 10.1590/0034-7167-2025-0275

**Published:** 2026-05-08

**Authors:** Davydson Gouveia Santos, Vanessa Martinhago Borges Fernandes, José Luís Guedes dos Santos, Fábio Silva Rosa, Morgana Oliveira Citolin, Mara Ambrosina de Oliveira Vargas

**Affiliations:** I Universidade Federal de Santa Catarina. Florianópolis, Santa Catarina, Brazil

**Keywords:** Forensic Nursing, Nursing Care, Forensic Sciences, Professional Qualification, Practical Nursing., Enfermería Forense, Atención de Enfermería, Ciencias Forenses, Cualificación Profesional, Enfermería Práctica.

## Abstract

**Objectives:**

to understand forensic nurses’ perspectives and direct advances in forensic nursing in Brazil.

**Methods:**

a qualitative study conducted through semi-structured online interviews between April and May 2023 with 22 forensic nurse specialists. The interviews were subjected to content analysis using Qualitative Data Analysis Miner software.

**Results:**

three thematic categories emerged: Dissemination and strengthening of forensic nursing education in undergraduate and graduate programs; Political involvement for the development of national regulations that enable forensic nursing practice in related services; Vacancies for forensic nursing and its inclusion in specialized services for identifying and combating violence.

**Final Considerations:**

the findings demonstrate the convergence of goals for strengthening forensic nursing in Brazil, highlighting the expansion of education, political articulation for national regulations and the creation of specific vacancies in specialized services to combat violence.

## RESUMEN

**Objetivos:**

comprender las perspectivas de las enfermeras forenses y guiar los avances en la enfermería forense en Brasil.

**Métodos:**

este estudio cualitativo se realizó mediante entrevistas semiestructuradas en línea entre abril y mayo de 2023 con 22 enfermeras forenses especialistas. Las entrevistas se sometieron a análisis de contenido mediante el *software Qualitative Data Analysis Miner*.

**Resultados:**

surgieron tres categorías temáticas: Difusión y fortalecimiento de la formación en enfermería forense en programas de pregrado y posgrado; Participación política para el desarrollo de normativa nacional que viabilice el ejercicio de la enfermería forense en servicios afines; Vacantes de enfermería forense y su inclusión en servicios especializados de identificación y combate a la violencia.

**Consideraciones Finales:**

los hallazgos demuestran la convergencia de objetivos para el fortalecimiento de la enfermería forense en Brasil, destacándose la expansión de la formación, la articulación política para la reglamentación nacional y la creación de plazas específicas en servicios especializados para el combate a la violencia.

## INTRODUCTION

Forensic nursing in Brazil has established itself as an essential field within the context of public healthcare and justice. It is defined as the integration of forensic sciences and nursing, aiming to collect, preserve, and document physical, psychological, and social evidence in criminal situations, especially those involving sexual, domestic, and other types of abuse. The role of forensic nurses is crucial, as these professionals are responsible for collecting evidence, preparing expert reports, and supporting criminal investigations, ensuring that evidence is preserved and properly analyzed within the judicial system^(1)^.

The development of forensic nursing in Brazil has a recent history, with notable advances in the last decade. However, the country still faces challenges related to specialized academic training, the implementation of forensic practices, and the integration of these professionals into healthcare services. Regarding the development of forensic nursing education, there are still gaps in nursing curriculums that do not adequately address the forensic aspects of the profession. The inclusion of forensic education in undergraduate programs, as well as the offering of graduate programs and continuing education, is essential for nurses’ training and the strengthening of the profession. Furthermore, the creation of partnerships between educational institutions, healthcare services, and the justice system is crucial to provide nursing students with practical training focused on the realities of forensic practice, fostering an interdisciplinary environment that unites healthcare, public safety, and legal professionals^(2)^.

Political strengthening for the development of forensic nursing in Brazil is another determining factor for the consolidation of this specialty. The country needs a clear regulatory framework that includes the development of public policies aimed at the training, recognition, and inclusion of forensic nurses in healthcare networks. In 2011, the Federal Nursing Council (COFEN) recognized forensic nursing as a specialty, but the practice still lacks greater legislative support to ensure full and structured coverage throughout the country^(3)^. Investing in incentive programs, regulating specialized courses, and creating support networks for professionals can strengthen the profession and ensure that victims of violence receive more comprehensive and specialized healthcare. Implementing public policies to strengthen this role should include greater integration between the healthcare and justice systems, as well as encouraging the development of national care protocols^(2)^.

The integration of these professionals into healthcare services is one of the main challenges facing forensic nursing in Brazil. Despite the importance of this role in caring for crime victims, especially in emergency units, healthcare services lack forensic nurses. The lack of infrastructure in hospitals and the scarcity of financial resources hinder the implementation of a specialized technical team, resulting in inadequate care for victims and hindering evidence collection. It is urgent that forensic nurses be more widely integrated into healthcare services so that they can work efficiently in documenting injuries and preserving evidence. Working collaboratively with other professionals, such as physicians, psychologists, and social workers, is essential to ensure quality multidisciplinary care^(4)^.

Furthermore, the inclusion of forensic nurses in healthcare facilities must be accompanied by continuing updates to protocols for treating victims of violence. Implementing unified protocols that address the physical, psychological, and social care of victims, in addition to evidence collection, could ensure that victims of violence are treated more effectively and that evidence in their cases is properly preserved^(5)^. Developing continuing training programs for healthcare professionals is essential to ensure everyone involved is prepared to handle the complexities of forensic care. Training in areas such as evidence collection, evidence preservation, and medical report writing is essential for forensic work to be carried out accurately and effectively^(4)^.

Despite recent advances in the consolidation of forensic nursing as a field of practice in Brazil, studies that systematically explore the perspectives of professionals who daily experience the challenges and potential of this specialty are still scarce. The national literature presents specific contributions on technical competencies, integration into healthcare services, and interprofessional coordination, but lacks prospective analyses that guide goals and strategies for strengthening the field in the coming decades. This gap highlights the need to investigate the perspectives of Brazilian forensic nurses to inform training policies, innovative practices, and scientific advances that consolidate the specialty nationally. Thus, the guiding question of this study was: what are forensic nurses’ perspectives for the advancement of the specialty in Brazil?

## OBJECTIVES

To understand forensic nurses’ perspectives and direct advances in forensic nursing in Brazil.

## METHODS

### Ethical aspects

This study followed all legal guidelines and prerogatives established by Resolutions 466/2012 and 510/2016 of the Brazilian National Health Council. It received a favorable opinion from the *Universidade Federal de Santa Catarina* Research Ethics Committee in December 2022. Participants consented to participate in the study by signing an online Informed Consent Form. To ensure anonymity, participants were identified by the abbreviation “FN” (forensic nurse), followed by a cardinal number in ascending order.

### Theoretical-methodological framework

This study adopts as a theoretical-methodological framework Resolution 556/2017 of COFEN, a regulation that recognizes and regulates forensic nursing’s work in Brazil^(6)^. This resolution establishes the technical, ethical, and legal competencies that guide professional practice in this specialty, defining responsibilities ranging from welcoming and caring for victims of violence to preserving remains and interacting with the health, justice, and public safety systems. Thus, it constitutes an essential normative framework for understanding the role and limits of forensic nursing practice in the national context, serving as a basis for interpreting the discourses analyzed in this research.

Based on Resolution 556/2017, this study anchors its analysis in a legal device that legitimizes forensic nursing practice, providing methodological parameters for reflection on its consolidation in Brazil^(6)^. Regulations not only guide professionals’ training and practice but also establish a convergence between the scientific, ethical, and social principles that underpin the specialty. Therefore, their use as a reference allows the findings of this investigation to be aligned with the guidelines that structure forensic nursing practice, strengthening analysis validity and contributing to the development of scientific knowledge in line with current regulatory frameworks.

### Study design

This qualitative study, with an exploratory approach, nationwide, was developed in accordance with COnsolidated criteria for REporting Qualitative research guidelines.

### Study setting

This research was carried out in a virtual environment, with national scope, initially through the completion of a digital form on Google Forms^®^ platform to characterize participants^(7)^ and demonstration of interest in participating in the synchronous online interview, conducted via the Google Meet^®^ digital platform with study participants.

### Data source

After completing ethical procedures and completing the digital form, participants were approached to participate in the study. Participant recruitment used the snowball technique, characterized as non-probability sampling. In this approach, referral chains served to identify potential research collaborators. Initially, the first participants were contacted based on a referral from the study’s lead author, and later, new participants were included through recommendations from the respondents themselves.

Nurses with a *lato sensu* specialization in forensic nursing and practical experience in nursing care or teaching in Brazil were included. Nurses with less than five years of nursing training, no experience providing care to people not experiencing violence, who did not respond to requests, or whose schedules conflicted with online interviews were excluded.

Twenty-two nurses participated in the semi-structured interviews, which were concluded using the theoretical data saturation criterion, achieving theoretical density caused by repetitions of content, without the emergence of new information or relevant analytical categories, ensuring consistency and depth of analysis, and avoiding redundancy^(8)^.

### Data collection and organization

The interviews were conducted in April and May 2023. A semi-structured interview guide was used to meet the study’s objectives. The following guiding questions formed the interview guide: what are your perspectives for Brazilian forensic nursing? What goals can be set for the next decade of Brazilian forensic nursing? The lead researcher conducted the interviews at a scheduled time with professionals, virtually and synchronously, via Google Meet^®^. Participants’ privacy was maintained, and the interviews were audio-recorded and lasted 20 minutes to 1 hour and 10 minutes, with an average of 32 minutes. The audio recordings of the interviews were transcribed verbatim.

### Data analysis

Data analysis was conducted using the thematic content analysis technique proposed by Bardin^(9)^, encompassing the pre-analysis, material exploration, data processing, and interpretation stages. Initially, a cursory reading of the interviews was performed to identify common aspects and enable a comparative analysis of discursive elements. Subsequently, in-depth readings were performed to capture information directly related to the study objectives. For this phase, the Qualitative Data Analysis Miner^(10)^ software, developed to assist in qualitative data management, coding, and analysis, was used. The study files were organized on the platform, enabling improved visualization of the formation of units of meaning and categories, facilitating a more fluid reading of participants’ statements and promoting continuous categorization of analyzed excerpts.

Thus, the data were explored and classified, identifying the recording units, understood as groupings of elements that were reorganized into categories and subsequently interpreted in light of the theoretical framework, in line with the study objective. Representative excerpts from the thematic categories are presented in the discussion of results. The study categories were defined based on the findings of the analysis, considering the theoretical framework, the research problem, and the proposed objective. Based on the analysis of the interviews, 22 recording units were identified during the coding process. After grouping by similarity, Chart 1 was constructed, which summarizes the categories, subcategories, and recording units.

**Chart 1:** Categories, subcategories, and registration units, Brazil, 2023

CATEGORIES	SUBCATEGORIES	RECORDING UNITS
Dissemination and strengthening of forensic nursing education in undergraduate and graduate programs with advancement of studies related to the area	Inclusion of forensic nursing in undergraduate studies	Need for basic knowledge about victim reception and referral
		Inclusion in undergraduate curricula as a form of early awareness
		Generalist training unprepared to deal with forensic demands
	Continuing education and graduate studies	Institutional awareness for specialized training
		Increase in the quantity and quality of graduate courses, avoiding a purely theoretical nature
		Graduate courses as an instrument for visibility and training in the field
	Scientific production and strengthening of research	Weakness of national research compared to other countries
		Need to expand scientific publications and internationalization
		Creation of specific master’s and doctorate programs in forensic nursing
Political involvement for the development of national regulations that enable forensic nursing practice in related services	Political mobilization and institutional articulation	Importance of political movement as a driver of change
		Need for coordination with parliamentarians and managers
		Political progress depends on greater action by the Federal Nursing Council
	Public policies and institutional resources	Creation of state commissions by the Federal Nursing Council as a mobilizing strategy
		Lack of public policies and specific resources to absorb professionals
	Insertion of forensic nursing in public safety policies	Capillarity of nursing in the territory as a potential to integrate public security
Vacancies for forensic nursing and its inclusion in specialized services for identifying and combating violence and in hospital emergency units	Public tenders and legal recognition of the function	Expectation of specific notices in scientific police and forensic medical institutes
		Desire for national and state competitions with vacancies for forensic nurses
		Financial difficulty without specific competitions
	Insertion into healthcare and safety services	Insertion of forensic nurses in health departments, primary care and emergencies
		Presence in hospitals to care for victims of violence
		Progressive inclusion in state competitions as an example for others
	Reference in care for victims of violence	Specialized action in sexual violence against women, children, the elderly and populations deprived of liberty

## RESULTS

Twenty-two forensic nurses participated in the interviews, the majority of whom were female (77.3%) and had a mean age of 42 years (SD=9.78; min=29; max=62). The majority considered themselves white (45.5%), were mostly married or in a stable union (45.5%), and were predominantly Catholic (68.2%). As for professional training, 63.6% of interviewees reported having between seven and 20 years of professional experience, and had, in addition to a specialization in forensic nursing, a *stricto sensu* degree (59.1%). Table 1 presents the educational profile of these professionals.

**Table 1:** Profile of forensic nursing training, Brazil, 2023

Variables		n	%
**Year of training in forensic nursing**	2015	1	4,5
	2018	5	22,8
	2019	7	31,8
	2021	3	13,6
	2022	5	22,8
	2023	1	4,5
	Total	22	100
**How did you acquire the title of forensic nursing specialist**	In-person specialization	9	40,9
	Distance learning	2	9,1
	Proof of qualifications from professional associations representing the field	11	50
	Total	22	100
**Institution that conferred the title of forensic nurse**	Brazilian Association of Forensic Nursing	4	18,2
	Brazilian Society of Forensic Nursing	7	31,8
	*Faculdade IDE - Universidade Cristo Redentor*	3	13,6
	Others	8	36,4
	Total	22	100

Among the study respondents, 12 (54.5%) were registered as forensic nursing specialists with the Regional Nursing Council (In Portuguese, *Conselho Regional de Enfermagem* - COREN), to which they were affiliated. Regarding their affiliation with representative bodies in the field, 11 (50%) were associated with the Brazilian Society of Forensic Nursing (In Portuguese, *Sociedade Brasileira de Enfermagem Forense* - SOBEF), six (27.3%) with the Brazilian Association of Forensic Nursing (In Portuguese, *Associação Brasileira de Enfermagem Forense* - ABEFORENSE), and five (22.7%) were not affiliated with representative bodies in the field.

The interviewees reported working in teaching and research (n=12), healthcare (n=10), management (n=8), and entrepreneurship (n=1), and some worked in more than one area simultaneously. They also possessed other nursing care, administrative, and educational specialties related to public health; mental health; clinical specialties in different stages of life (neonatology, pediatrics, obstetrics and gynecology, and gerontology); emergency and intensive care; healthcare management; hospital and nursing administration and auditing; occupational health nursing; professional education and bioethics; among others. These professionals apply forensic knowledge to various areas of nursing practice.

Based on the analysis of the interviews, three thematic categories emerged: Dissemination and strengthening of forensic nursing education in undergraduate and graduate programs with the advancement of studies related to the area; Political involvement for the development of national regulations that enable forensic nursing practice in related services; Vacancies for forensic nursing and its inclusion in specialized services for identifying and combating violence and in hospital emergency units.

### Dissemination and strengthening of forensic nursing education in undergraduate and graduate programs with advancement of studies related to the area

In the first thematic category, interviewees emphasize the urgent need to include forensic nursing in undergraduate and graduate curricula as a way to ensure that nurses, from their initial training, possess basic knowledge of forensic case management, such as welcoming victims of violence and conducting appropriate referrals. As one interviewee highlighted, a basic understanding of forensic practice could provide a solid foundation for nursing professionals, even those without specialization, to act appropriately in situations of violence.

*It’s important to change this entire curricular dynamic at universities. Professors must be aware of their field and stay up-to-date on cross-cutting topics related to forensic nursing and what’s being discussed. You don’t need to be a forensic nurse to have the basic knowledge a generalist nurse needs about their duty under the code of ethics, referrals, notification, and, when necessary, filing a complaint. You don’t need to be a forensic nurse to perform this action. Professionals, having at least a general understanding of reception and guidance in cases related to forensic issues in their training, will already be helping and will be supported by the basic knowledge acquired during their training at university.* (FN03)

*We can’t work on something if people don’t know it. Incorporating forensic nursing knowledge into undergraduate studies is crucial. By engaging with it during your initial and original training, you’ll be able to learn-even if you don’t become a forensic nurse later-about some of the related care you need to take. I advocate for incorporating forensic nursing knowledge into undergraduate studies.* (FN11)

*Introduce forensic nursing into the university curriculum, because when nursing professionals become professionals and graduate with qualifications to work, they leave very unprepared, and they can even be legally harmed sometimes* [...] *I know it’s a difficult thing, that universities don’t want it, they even think it’s beautiful, really cool, but not in the curriculum, it’s unlikely to be included as extracurricular content.* (FN08)

*I always say in my lectures: we need to raise awareness in institutions, whether public or private so that forensic nursing is present across all disciplinary topics that encompass violence, or as forensic nursing as a topic with our competencies so that we can raise awareness among new trainees and our graduates so they have a sense of what the specialty entails. On the other hand, we need to solidify lato sensu programs, providing them with robust content, created by experienced professionals, who truly and rightfully provide the training that professionals need to avoid encroaching on others’ areas and not neglecting their own field. When I say “the other’s field”, I mean the other professional’s field, another specialty-the nurse’s own field.* (FN18)

The need to expand forensic nursing education from undergraduate to *stricto sensu* graduate courses through robust scientific studies with methodological rigor became evident so that forensic nursing can be strengthened by expanding scientific knowledge.

*The starting point is training* [...] *increasing quantity and quality as well, because when you increase quantity, you run the risk of losing quality, of completing a completely theoretical graduate program, without any experience in any field, not even a trace of violence, not even prison, not even disaster, and being left with that paper-based title, without any practical experience, like I know nurses who are ICU specialists and don’t know what an infusion pump is. It’s about this, beyond quantity, qualifying, training.* (FN13)

*Regarding graduate programs, the trend is for others to begin emerging. The more specialized professionals, the greater the field’s visibility. There is no specific program for forensic nursing* [*stricto sensu*]*. Graduate programs should be interested in training well-qualified professionals who can direct specific studies in the field and train other professionals with excellence. I believe this is one of the issues that can advance with the development of research.* (FN03)

*I believe we can contribute significantly to various aspects of forensic nursing in Brazil, and that it’s impossible not to link several social challenges to our work without conducting research. The problem is that we need support and resources for this. We need to make significant progress in research. I think forensic nursing research is very weak compared to other countries. We need to advance in studies, in the types of studies we conduct. One of the best ways for us to demonstrate our importance is by presenting research-more robust studies, with a large population-so that government authorities can see the importance and understand how poor the service is.* (FN09)

[...] *just as we mirror ourselves in American forensic nursing, we can take our practice from here to there as well, not only there, but to other developing countries* [...] *we need to write more, we need to be more scientific.* (FN15)

[...] *it’s a path we need to tackle to create, beyond a single line of research, a master’s degree, a doctorate specifically for forensic nurses. Because, when we have a specific master’s degree, and not just a single line of research, I’m sure we’ll have a lot of scientific production, and I believe that scientific production is unquestionable. It should be robust scientific production, with field research, multicenter, and not just literature reviews, as we’ve seen* [...] *it’s the universities that need to tackle this. Until we have this support from universities, especially public ones, it will take a while to get there. We will get there. No one will stand still halfway, but I say the university, because it brings knowledge with it; it brings research; it brings criticism; it even criticizes itself when it’s about to do research: “This isn’t the way, no, this isn’t for you, no, let’s do another one”, and then we’ll work on the stitches to get there.* (FN18)

### Political involvement for the development of national regulations that enable forensic nursing practice in related services

In the second thematic category, interviewees highlighted the importance of a coordinated political movement that involved not only nursing professionals, but also COFEN, parliamentarians and public managers, for the creation of laws that ensure the role of forensic nurses in public tenders and in scientific police systems, Institutes of Legal Medicine (ILMs) and other services that serve victims of violence.

*Without the political movement of those who are being formed, nothing will happen, because it is only with political movement that things happen. I often say that any administrative action is, first and foremost, political. SOPs, before being administrative, are political* [...]. (FN02)

*I see a boom, especially in the last two years, and significant progress. To advance further, we need to establish fields of expertise. We need to be able to discuss this politically with representatives, senators, and council members-those people who can help, who wield political power, who can also bring the specialty into the field-so that we can move beyond the theoretical realm and begin to see how it works in practice here in Brazil. I believe the progress now will be progressive, only moving forward. There’s no stopping forensic nursing in Brazil; we’re going to become a benchmark in Latin America. I don’t see other Latin American countries moving the way we have here to advance the specialty.* (FN04)

[...] *we’ve already made progress; today we have the CBO, which has the specialty. To make further progress, we need a more aggressive policy, including from COFEN. Pressure the legislature, pressure leaders to actually create the role of forensic nurse in public service exams, to develop expertise that can be used in the prison system. For us to truly move forward, we need this political action and a more aggressive policy from the Federal Council* [...] *otherwise, we’ll be left with only dreams and projects.* (FN05)

Having highlighted the need for this political articulation as a fundamental strategy for strengthening the field, COFEN’s role in creating state commissions is highlighted as a mobilizing action. Concern is expressed about the lack of specific public policies and adequate resources for the inclusion of forensic nurses in institutions, which hinders the utilization of these professionals. Furthermore, participants reinforce nursing’s capillarity throughout the country, advocating for the inclusion of forensic nursing in public safety policies, in coordination with the Ministries of Health and Defense, as a way to expand the fight against violence and improve victim care.

*The political issue will be our great support, because, currently, COFEN has started to form committees in several states* [...]. (FN10)

*There is a lack of public policies to provide resources to absorb forensic nurses and for institutions to appropriate these professionals who are graduating.* (FN09)

*Nursing is a capable science; it has truly qualified professionals. We’re present throughout the country. We don’t have a courthouse in every municipality, but we do have nurses in every municipality. We’re present from one end to the other. Forensic nursing needs to be incorporated into public safety policies, working alongside the Ministry of Health, the Ministry of Defense, and their state and municipal departments in combating violence.* (FN10)

### Vacancies for forensic nursing and its inclusion in specialized services for identifying and combating violence and in hospital emergency units

In the third thematic category of this study, professionals present the need for a public competition with specific vacancies for the area, as well as some possibilities for inserting this professional to provide assistance to people who have experienced some situation of violence, not just as a complementary service.

*One of the biggest gains will be when you look at a forensic police announcement and see forensic nursing inserted into interdisciplinary areas, or within a hospital, a service partnering with the Forensic Medical Institute or the Death Verification Service, partnerships with the judiciary, with the police station. So, with these partnerships, you’ll begin to gain credibility and greater visibility* [...] *the issue of public service exams is one of the greatest desires of the societies themselves* [SOBEF, ABEFORENSE] *to have exams focused on this area, because we see this catastrophic issue happening in the country with the increase in violence. I hope that in the future this can gain greater visibility.* (FN03)

*In the next decade, I envision more forensic nurses trained and registered with their boards so that when a list is compiled, there will be at least 20 in one state, 10 in another, 15 in another. A national competition for forensic nurses could be held; competitions could be held within the healthcare sector, with states opening competitions for ICU nurses, emergency nurses, and forensic nurses. Simplified selection processes for forensic nurses, especially in institutions that are references for the care of victims of violence, for the long-awaited career within forensic institutes, forensic science, forensic police, police competitions, and courts. There’s a lot of work to be done to make this happen. Today, we aspire to it, we dream of it. Who knows, maybe it will happen in the next decade. There are still doors we’ll knock on a lot, but they will open. We need to occupy these existing spaces. People just don’t realize the benefits to society that occupying these spaces will bring.* (FN07)

[...] *until we have public competitions for forensic nurses, we won’t have many opportunities, we won’t have much success in this field. I study because I enjoy it; it’s a field that gives me pleasure. It’s strange to talk about violence, death, and disasters while talking about pleasure, but it’s something that gives me satisfaction in helping those most in need. But I know that financially, I’ll hardly see any return from this field.* (FN16)

The survey results reveal a set of expectations and proposals regarding the effective integration of forensic nurses into health and public safety structures. Participants emphasize the need to integrate these professionals into Municipal and State Health Departments, promoting matrix support for primary care, emergency, and urgent care teams, especially in the care of victims of violence in acute situations. The desire for forensic nursing to become a reference in specialized services addressing sexual violence against women, children, older adults, and people deprived of liberty stands out. Interviewees also emphasize the transformative role that forensic nursing can play within services, inspiring other states to adopt similar initiatives and contributing to improving care and access to justice for victims.

*The inclusion of forensic nurses in Municipal and State Health Departments, creating a matrix of primary care teams for emergency and urgent care teams. We should have forensic nurses serving victims of violence in acute situations, which are in the emergency and urgent care services, with a specialized room. We should truly be a reference in the service of sexual violence against women, children, and the elderly in the prison system. We should succeed in creating the position, especially in ILMs. This part is perhaps the most complex and time-consuming: having a public selection process with a specific position for us. But inclusion in the departments, in the school health program, and in emergency and urgent care units is one way forward.* (FN14)

*I want to see all civil police competitions offer forensic nursing positions, and these network partnerships grow more and more, and become something that exists within hospitals. For instance, here at the hospital, I would really like to have a forensic nurse position available for emergency care* [...] *there are some nurses who care for victims of violence and sometimes they don’t even know what to do, how to approach, how to refer. I have these expectations.* (FN17)

*Look, I was looking at the Pernambuco competition, where COREN requested the inclusion of forensic nurses as experts. I thought it was fantastic. I think that’s it, a painstaking effort. If one state manages to approve this, it’s a no-brainer for another state. So, from the moment forensic nurses start making a difference in scientific forensics, this will be a mirror, a good example for other states to include our specialty, with our role, carrying out very beautiful, very well-done social work, because that’s what we really created the specialty for: to make a difference.* (FN19)

## DISCUSSION

Forensic nursing in Brazil is an emerging field that presents significant challenges both in terms of academic training and political and legislative recognition, as well as the inclusion of forensic nurses in specialized services.

Academic training in forensic nursing is one of the pillars for strengthening this specialty in Brazil. A previous study highlights the importance of integrating this knowledge across university curricula^(2,11)^. Forensic nursing is not restricted to experts’ specific practice, but encompasses essential skills for managing victims of violence, reporting crimes and dealing with trauma situations^(12)^.

The inclusion of forensic content in basic nursing training, such as the code of ethics, mandatory reporting, and support for victims of violence, is essential to ensure that all professionals, even those not specialized, know how to proceed in critical situations. Although undergraduate programs cover topics related to health and clinical care, forensic nursing is still often treated as a marginal topic or, when addressed, in extracurricular modules^(2,11)^. This reality reflects a significant gap in Brazilian nurses’ training, requiring a change in university curricula so that generalist nurses also receive minimal preparation, even if they do not specialize in the area.

The need for more qualified graduate programs, with a more practical rather than theoretical focus, was also a point discussed by the interviewees. Increasing the number of specialists is important, but the quality of training is even more crucial to avoid the creation of professionals who, despite holding specialist qualifications, lack the practical experience necessary to work in the field^(13,14)^. It is essential that specialized training includes real experiences in scenarios of violence, disasters and investigations, in order to prepare nurses for the challenges and complexities that arise in these situations^(15,16)^.

Furthermore, the integration between theory and practice in graduate courses is essential to ensure that nurses not only understand legal and forensic concepts, but also have the ability to identify evidence and understand the particularities of care for victims of violence^(17,18)^. This reinforces the need for more robust studies that provide a solid scientific basis for forensic nursing, especially in Brazil, where investments in scientific research in this area are still scarce compared to other countries^(19)^.

In the United States, forensic nursing is a well-established field in both academic training and clinical practice. There, undergraduate and graduate programs in forensic nursing are well-established, with dedicated programs that cover topics such as evidence collection, injury documentation, judicial practice, and victim health. Furthermore, many states require forensic nurses to undergo additional certifications to work with victims of sexual assault, within an integrative practice model between the healthcare system and the justice system. This helps to train professionals better prepared to work at the intersection of nursing, public health, and law^(17,18,20)^.

This model could serve as a benchmark for Brazil, where the integration of forensic nursing into university curricula is still limited. As suggested by interviewees, initial training could be more robust, with a greater focus on forensic competencies, and could be complemented by graduate programs offering formal specialization^(14)^.

Another key factor in advancing forensic nursing in Brazil concerns professionals’ political involvement in this field. The creation of national regulations that establish the role of forensic nurses in healthcare services, such as hospitals and emergency units, as well as in public safety and justice, is crucial to ensuring the inclusion of these professionals in the job market.

The role of forensic nurses is essential not only for victim care, but also to ensure the validation of medical reports, collect evidence that can be used in criminal investigations and provide testimony in court^(21)^. In this regard, political action and pressure from entities, in this case, representatives of forensic nursing in Brazil, such as COFEN, SOBEF, and ABFORNSE, are essential to ensure that forensic nursing is included as a recognized specialty and has its role institutionalized in different areas, such as public health, security, and justice^(22)^.

The lack of a solid public policy to encourage the hiring of forensic nurses is a barrier to the expansion of this specialty in Brazil. The lack of financial resources and the absence of public competitions with specific positions for the field hinder these professionals’ access to specialized spaces. As one of the interviewees pointed out, political pressure must intensify for forensic nursing to become part of public health policy, especially in areas that combat violence, such as sexual violence, domestic violence, and crimes against children and older adults^(23-26)^.

In the United States and the United Kingdom, forensic nurses have stronger legal support, with specific regulations for the profession and a clear role within the justice system. The British healthcare system, for instance, has invested in continuing training programs for forensic nurses, creating public policies to include forensic nursing in the care of victims of sexual violence, domestic violence, and other forms of abuse. In the United Kingdom, forensic nurses are responsible for collecting evidence, conducting forensic interviews, and even acting as witnesses in court. This integration of nursing into the justice system is protected by a series of regulations, which ensures the quality of care and the legal security of professionals and victims^(27-29)^.

In Brazil, the lack of clear public policies and political support for the creation of specific competitions for forensic nurses is seen as a significant obstacle to forensic nursing expansion. However, the growing mobilization of organizations such as COFEN and the inclusion of forensic nursing on the agendas of some political spheres may be the first steps toward institutionalizing this practice in the national healthcare and justice systems.

The inclusion of specific positions for forensic nurses, especially in services such as medical examiners’ offices, referral hospitals, police stations, and other security and justice institutions, was a recurring demand in the interviews. Many interviewees expressed a desire to see forensic nursing integrated into public service exams, with specific positions for the field.

Creating positions for forensic nurses in public institutions and the prison system could provide greater credibility to these professionals’ work and also ensure that victims of violence receive more efficient and qualified care^(30-32)^. Forensic nurses have the ability to collect forensic evidence and provide emergency medical care while ensuring the preservation of the chain of custody of evidence. This professional, therefore, would be an essential element in criminal investigations and the judicial process, playing a role in both healthcare and justice^(29,33-35)^.

The inclusion of these professionals in emergency units and in the care of victims of sexual violence and domestic violence is seen as a way to improve quality of care, providing technical support to medical and police teams^(27,36)^. However, as pointed out by the interviewees, the lack of public tenders with specific vacancies and infrastructure in hospitals and ILMs is a significant obstacle to the insertion of forensic nurses in these institutions.

International experiences, such as those in Australia, can serve as an example for Brazil in this regard. The inclusion of forensic nurses in emergency response teams, such as on the front lines of care for victims of sexual violence and traumatic accidents, is a well-established practice in the country. Furthermore, Australian forensic nurses frequently collaborate with police forces and other government agencies in criminal investigations, demonstrating the importance of interdisciplinarity. The creation of specific positions for forensic nurses in public tenders is also a reality in Australia, where the government recognizes the need for professionals trained to work in situations of violence and tragedy^(22,37,38)^.

Looking ahead to the next decade, forensic nursing in Brazil is poised to consolidate itself as a strategic specialty at the interface between health, justice, and public safety. Advances in teaching and research are expected to translate into greater professional qualifications, with the definitive inclusion of the topic in undergraduate curricula and the expansion of *lato sensu* and *stricto sensu* graduate programs capable of producing robust scientific knowledge applicable to the Brazilian reality. At the same time, the category’s political mobilization is expected to be strengthened, with the engagement of representative entities and regulatory bodies in the development of national regulations that ensure forensic nurses’ work in different institutional spheres.

This movement should encourage the creation of public tenders and the opening of specific positions for the specialty in healthcare services, emergency hospital units, medical examiners’ offices, and specialized police stations, responding more effectively to the growing demands of combating violence. Thus, the next decade is shaping up to be a period of expansion, institutionalization, and social recognition for forensic nursing in Brazil, with the potential to positively impact both the quality of care provided to victims and the strengthening of public security and justice policies.

### Study limitations

One of the limitations of this study is methodological, considering that the lead author was unable to provide feedback on the interviews for participants’ respective conferences. Another limitation was the number of participants, especially since this is a nationwide study in the largest health field, nursing. However, the study highlights the need to further expand and disseminate this field of knowledge. The results presented highlight the current profile of professionals working and committed to a growing area of nursing, in addition to offering some concrete perspectives for the specialty. The participation of dedicated professionals enhances the creation of strategies for the development of the field, in addition to providing support for future initiatives to apply forensic nursing in healthcare services.

### Contributions to nursing, health and public policy

From participants’ perspective, this study reinforces the importance of forensic nursing knowledge being present from generalist training to graduate studies, enabling the advancement of studies and strengthening the field. This fosters the development of cognitive potential and skills in nursing students and professionals, as it facilitates an understanding of the phenomena and dimensions addressed in this specialty, as well as their professional responsibilities in these contexts. Political engagement is essential for the development of regulations that allow forensic nursing practice in specialized and related services. This strengthening could lead to the implementation of forensic nursing positions in public healthcare services and their role in hospital emergency departments.

## FINAL CONSIDERATIONS

The analysis of results showed that forensic nursing in Brazil, although still in the process of consolidation, has clear prospects for strengthening and expansion over the next decade. The findings revealed a convergence of strategic goals among professionals in the field, organized around three main axes: the dissemination and improvement of specialty education from undergraduate to *stricto sensu* graduate programs, with a view to robust and applicable scientific production; political and institutional engagement as an essential path to creating national regulations that legitimize and regulate forensic nursing practice; and the formal inclusion of these professionals in public service exams and specialized services, especially in combating violence in hospital settings, forensic medical institutions, and public security systems.

These goals converge toward building a more solid professional identity, capable of responding to emerging social demands related to violence and human rights violations, while positioning forensic nursing as a strategic interface between health, justice, and security. Therefore, we conclude that the findings of this research project a scenario of greater institutionalization, recognition, and social impact for forensic nursing in Brazil over the next decade, provided it is accompanied by investments in training, research, public policies, and the creation of specific spaces for action that ensure not only the visibility but also the effectiveness of this specialty’s contribution to society.

## Data Availability

The research data are available within the article.
