# Supplementary material for: Brazilian forensic nursing: convergence of goals for the specialty’s next decade
Source: Rev Bras Enferm. 2026 May 8;79:e20250275. doi: 10.1590/0034-7167-2025-0275 (PMC13169630; doi:10.1590/0034-7167-2025-0275)
Supplement: Translation

# Translation: Enfermagem forense brasileira: convergência de metas para próxima década da especialidade

**DOI:** 10.1590/0034-7167-2025-0275pt

**Authors:** Davydson Gouveia Santos, Vanessa Martinhago Borges Fernandes, José Luís Guedes dos Santos, Fábio Silva Rosa, Morgana Oliveira Citolin, Mara Ambrosina de Oliveira Vargas

**Affiliations:** I Universidade Federal de Santa Catarina. Florianópolis, Santa Catarina, Brasil

**Keywords:** Enfermagem Forense, Cuidados de Enfermagem, Ciências Forenses, Qualificação Profissional, Enfermagem Prática.

## Abstract

**Objetivos:**

conhecer as perspectivas dos enfermeiros forenses e direcionar os avanços da enfermagem forense no Brasil.

**Métodos:**

estudo qualitativo, realizado por meio de entrevista semiestruturada *online*, entre abril e maio de 2023, com 22 enfermeiros especialistas em enfermagem forense. As entrevistas foram submetidas à análise de conteúdo com apoio do *software Qualitativa Data Analysis Miner*.

**Resultados:**

emergiram três categorias temáticas: Disseminação e fortalecimento do ensino da enfermagem forense na graduação e pós-graduação; Envolvimento político para o desenvolvimento de normativas nacionais que viabilizem a prática da enfermagem forense nos serviços relacionados; Vagas para enfermagem forense e sua inserção nos serviços especializados de identificação e combate da violência.

**Considerações Finais:**

os achados evidenciam a convergência de metas para o fortalecimento da enfermagem forense no Brasil, destacando a ampliação do ensino, a articulação política para normativas nacionais e a criação de vagas específicas em serviços especializados de enfrentamento da violência.

## INTRODUÇÃO

A enfermagem forense no Brasil tem se consolidado como uma área essencial dentro do contexto da saúde pública e da justiça, sendo definida como a integração das ciências forenses e da enfermagem, com o objetivo de coletar, preservar e documentar evidências físicas, psicológicas e sociais em situações de crimes, principalmente os que envolvem violência sexual, doméstica e outros tipos de abusos. A atuação do enfermeiro forense é crucial, visto que esses profissionais são responsáveis pela coleta de provas, elaboração de laudos periciais e apoio à investigação criminal, garantindo que as evidências sejam preservadas e corretamente analisadas dentro do sistema judicial^(1)^.

O desenvolvimento da enfermagem forense no Brasil tem um histórico recente, com avanços notáveis na última década. Porém, o país ainda enfrenta desafios relacionados à formação acadêmica especializada, à execução das práticas forenses e à inserção desses profissionais nos serviços de saúde. Em relação ao desenvolvimento do ensino da enfermagem forense, ainda existem lacunas no currículo de cursos de enfermagem que não abordam adequadamente os aspectos forenses da profissão. A inclusão do ensino forense nos cursos de graduação, assim como a oferta de programas de pós-graduação e capacitação continuada, é essencial para a capacitação de enfermeiros e o fortalecimento da profissão. Além disso, a criação de parcerias entre instituições de ensino, serviços de saúde e justiça é fundamental para proporcionar aos estudantes de enfermagem uma formação prática voltada à realidade da atuação forense, promovendo um ambiente interdisciplinar que une profissionais de saúde, segurança pública e direito^(2)^.

O fortalecimento político para o desenvolvimento da enfermagem forense no Brasil é outro fator determinante para consolidação dessa especialidade. O país necessita de um marco regulatório claro e que envolva a construção de políticas públicas voltadas à capacitação, ao reconhecimento e à inserção dos enfermeiros forenses nas redes de atendimento à saúde. Em 2011, o Conselho Federal de Enfermagem (COFEN) reconheceu a enfermagem forense como especialidade, mas a prática ainda carece de um maior apoio legislativo para garantir uma atuação plena e estruturada em todo o território nacional^(3)^. O investimento em programas de incentivo, a regulamentação de cursos especializados e a criação de redes de apoio aos profissionais são ações que podem viabilizar o fortalecimento da profissão, além de garantir que as vítimas de violência recebam um atendimento de saúde mais completo e especializado. A implementação de políticas públicas para fortalecer essa atuação deve incluir uma maior integração entre os sistemas de saúde e justiça, além do incentivo ao desenvolvimento de protocolos nacionais de atendimento^(2)^.

A inserção desses profissionais nos serviços de saúde é um dos principais desafios que a enfermagem forense enfrenta no Brasil. Apesar da relevância dessa atuação no atendimento às vítimas de crimes, especialmente em unidades de urgência e emergência, os serviços de saúde não possuem enfermeiros forenses. A falta de estrutura nos hospitais e a escassez de recursos financeiros dificultam a implementação de uma equipe técnica especializada, o que resulta em um atendimento inadequado às vítimas, prejudicando a coleta de evidências. É urgente que os enfermeiros forenses sejam inseridos de maneira mais ampla nos serviços de saúde, para que possam atuar de forma eficiente na documentação de lesões e na preservação das provas. A atuação integrada com outros profissionais, como médicos, psicólogos e assistentes sociais, é fundamental para garantir um atendimento multidisciplinar de qualidade^(4)^.

Além disso, a inserção dos enfermeiros forenses nas unidades de saúde deve ser acompanhada de uma atualização constante dos protocolos de atendimento às vítimas de violência. A implementação de protocolos unificados, que contemplem o cuidado físico, psicológico e social das vítimas, além da coleta de evidências, poderia garantir que as vítimas de violência fossem atendidas com mais eficácia e que as provas de seus casos fossem preservadas de maneira adequada^(5)^. A criação de programas de treinamento contínuo para os profissionais de saúde é essencial para assegurar que todos os envolvidos estejam preparados para lidar com a complexidade do atendimento forense. O treinamento em áreas como coleta de provas, preservação de evidências e redação de laudos médicos é imprescindível para que a atuação forense seja realizada com precisão e eficácia^(4)^.

Apesar dos avanços recentes na consolidação da enfermagem forense como campo de atuação no Brasil, ainda são escassos os estudos que exploram de forma sistemática as perspectivas dos profissionais que vivenciam cotidianamente os desafios e as potencialidades dessa especialidade. A literatura nacional apresenta contribuições pontuais sobre competências técnicas, inserção nos serviços de saúde e articulação interprofissional, mas carece de análises prospectivas que orientem metas e estratégias para o fortalecimento da área nas próximas décadas. Essa lacuna evidencia a necessidade de investigar a visão dos enfermeiros forenses brasileiros, de modo a subsidiar políticas de formação, práticas inovadoras e avanços científicos que consolidem a especialidade no cenário nacional. Assim, a questão norteadora deste estudo foi: quais as perspectivas dos enfermeiros forenses para o avanço da especialidade no Brasil?

## OBJETIVOS

Conhecer as perspectivas dos enfermeiros forenses e direcionar os avanços da enfermagem forense no Brasil.

## MÉTODOS

### Aspectos éticos

Este estudo seguiu todas as diretrizes e prerrogativas legais estabelecidas pelas Resoluções n^o^ 466/2012 e n^o^ 510/2016 do Conselho Nacional de Saúde. Obteve parecer favorável pelo Comitê de Ética em Pesquisa da Universidade Federal de Santa Catarina em dezembro de 2022. Os participantes manifestaram consentimento à participação do estudo mediante assinatura do Termo de Consentimento Livre e Esclarecido *online*. Para garantir o anonimato, os participantes foram identificados pela abreviatura “EF” (enfermeiro forense), seguido de um número cardinal em ordem crescente.

### Referencial teórico-metodológico

O presente estudo adota como referencial teórico-metodológico a Resolução nº 556/2017 do COFEN, normativa que reconhece e regulamenta a atuação da enfermagem forense no Brasil^(6)^. Tal resolução estabelece as competências técnicas, éticas e legais que orientam o exercício profissional nessa especialidade, definindo atribuições que vão desde o acolhimento e cuidado às vítimas de violência até a preservação de vestígios e interlocução com os sistemas de saúde, justiça e segurança pública. Assim, constitui-se em marco normativo essencial para compreender a inserção e os limites de atuação do enfermeiro forense no cenário nacional, servindo como base para interpretação dos discursos analisados nesta pesquisa.

Ao se fundamentar na Resolução nº 556/2017, este estudo ancora sua análise em um dispositivo legal que legitima a prática da enfermagem forense, fornecendo parâmetros metodológicos para a reflexão sobre sua consolidação no Brasil^(6)^. A normativa não apenas direciona a formação e a prática dos profissionais, mas também estabelece uma convergência entre os princípios científicos, éticos e sociais que sustentam a especialidade. Dessa forma, sua utilização como referencial permite alinhar os achados desta investigação com as diretrizes que estruturam o exercício da enfermagem forense, fortalecendo a validade da análise e contribuindo para a construção de conhecimento científico em consonância com os marcos regulatórios vigentes.

### Tipo de estudo

Estudo qualitativo, com abordagem exploratória, de abrangência nacional, desenvolvido de acordo com as diretrizes do *COnsolidated criteria for REporting Qualitative research*.

### Cenário do estudo

Pesquisa realizada em ambiente virtual, com abrangência nacional, inicialmente por meio do preenchimento de um formulário digital na plataforma do *Google Forms* ^®^ para caracterização dos participantes^(7)^ e demonstração de interesse em participar da entrevista *online* síncrona, realizada via plataforma digital *Google Meet^®^* com os participantes do estudo.

### Fonte de dados

Após trâmites éticos e preenchimento do formulário digital, os participantes foram abordados para participação do estudo. Para recrutamento dos participantes, utilizou-se a técnica *snowball*, caracterizada como uma amostragem não probabilística. Nessa abordagem, cadeias de referência serviram para identificar possíveis colaboradores da pesquisa. Inicialmente, os primeiros participantes foram contatados a partir de indicação realizada pelo autor principal do estudo e, posteriormente, novas inclusões ocorreram por meio de recomendações dos próprios respondentes.

Foram incluídos enfermeiros com especialização *lato sensu* em enfermagem forense, com experiência prática assistencial ou docência em território nacional. Foram excluídos enfermeiros que possuíam menos de cinco anos de formação em enfermagem, sem experiência assistencial com pessoas sem situação de violência, que não deram retorno ou com incompatibilidade de agenda para realização das entrevistas *online*.

Participaram das entrevistas semiestruturadas 22 enfermeiros, encerrando-as através do critério de saturação teórica dos dados, chegando ao adensamento teórico causado pelas repetições de conteúdos, sem o surgimento de novas informações ou categorias analíticas relevantes, garantindo consistência e profundidade das análises, e evitando redundância^(8)^.

### Coleta e organização de dados

As entrevistas foram realizadas nos meses de abril e maio de 2023. Para tal fim, foi utilizado roteiro semiestruturado para atender ao objetivo deste estudo. As seguintes questões norteadoras compuseram o roteiro: quais as tuas perspectivas para a enfermagem forense brasileira? Quais metas podem ser traçadas para próxima década da enfermagem forense brasileira? As entrevistas foram realizadas pelo pesquisador responsável em horário agendado com os profissionais, de maneira virtual síncrona, via plataforma do *Google Meet^®^*. Manteve-se a privacidade dos participantes, e as entrevistas foram audiogravadas e com duração de 20 minutos a uma hora e 10 minutos, com média de 32 minutos. Os áudios das entrevistas foram transcritos na íntegra.

### Análise de dados

A análise de dados foi conduzida por meio da técnica de análise de conteúdo temática, proposta por Bardin^(9)^, contemplando as etapas de pré-análise, exploração do material, tratamento de dados e interpretação. Inicialmente, realizou-se leitura flutuante das entrevistas com o objetivo de identificar aspectos comuns e permitir uma análise comparativa dos elementos discursivos. Em seguida, foram efetuadas leituras aprofundadas, visando captar informações diretamente relacionadas aos objetivos do estudo. Para esta fase, empregou-se o *software Qualitativa Data Analysis Miner* ^(10)^, desenvolvido para auxiliar na gestão, codificação e análise de dados qualitativos. Os arquivos do estudo foram organizados na plataforma, possibilitando uma visualização aprimorada da formação das unidades de sentido e das categorias, facilitando uma leitura mais fluida das falas dos participantes e promovendo uma categorização contínua dos trechos analisados.

Dessa forma, os dados foram explorados e classificados, identificando-se as unidades de registro, entendidas como agrupamentos de elementos que foram reorganizados em categorias e, posteriormente, interpretados à luz do referencial teórico, em consonância com o objetivo do estudo. Trechos representativos das categorias temáticas são apresentados na discussão dos resultados. As categorias do estudo foram definidas a partir dos achados da análise, considerando o referencial teórico, o problema de pesquisa e o objetivo proposto. A partir da análise das entrevistas, foram identificadas 22 unidades de registro durante o processo de codificação. Após os agrupamentos por similaridade, construiu-se o Quadro 1, que sintetiza as categorias, subcategorias e unidades de registro.

**Quadro 1:** Categorias, subcategorias e unidades de registro, Brasil, 2023

CATEGORIAS	SUBCATEGORIAS	UNIDADES DE REGISTRO
Disseminação e fortalecimento do ensino da enfermagem forense na graduação e pós-graduação com avanço dos estudos relacionados à área	Inclusão da enfermagem forense na graduação	Necessidade de conhecimento básico sobre acolhimento e encaminhamento de vítimas
		Inserção nos currículos de graduação como forma de sensibilização precoce
		Formação generalista despreparada para lidar com demandas forenses
	Formação continuada e pós-graduação	Sensibilização institucional para a formação especializada
		Ampliação da quantidade e da qualidade das pós-graduações, evitando caráter apenas teórico
		Pós-graduações como instrumento de visibilidade e capacitação da área
	Produção científica e fortalecimento da pesquisa	Fragilidade das pesquisas nacionais em comparação a outros países
		Necessidade de ampliar publicações científicas e internacionalização
		Criação de mestrado e doutorado específicos em enfermagem forense
Envolvimento político para o desenvolvimento de normativas nacionais que viabilizem a prática da enfermagem forense nos serviços relacionados	Mobilização política e articulação institucional	Importância do movimento político como motor de mudanças
		Necessidade de articulação com parlamentares e gestores
		Avanço político depende de maior atuação do Conselho Federal de Enfermagem
	Políticas públicas e recursos institucionais	Criação de comissões estaduais pelo Conselho Federal de Enfermagem como estratégia mobilizadora
		Ausência de políticas públicas e de recursos específicos para absorver os profissionais
	Inserção da enfermagem forense nas políticas de segurança pública	Capilaridade da enfermagem no território como potencial para integrar segurança pública
Vagas para enfermagem forense e sua inserção nos serviços especializados de identificação e combate da violência e nas emergências das unidades hospitalares	Concursos públicos e reconhecimento legal da função	Expectativa de editais específicos em polícia científica e institutos médicos legais
		Desejo de concursos nacionais e estaduais com vagas para enfermeiro forense
		Dificuldade financeira sem concursos específicos
	Inserção nos serviços de saúde e segurança	Inserção do enfermeiro forense nas secretarias de saúde, atenção primária e emergências
		Presença em hospitais para atendimento de vítimas de violência
		Inclusão progressiva em concursos estaduais como exemplo para outros
	Referência no atendimento às vítimas de violência	Atuação especializada em violência sexual, contra mulheres, crianças, idosos e populações privadas de liberdade

## RESULTADOS

Participaram das entrevistas 22 enfermeiros forenses, sendo a maioria do sexo feminino (77,3%) e com média de idade de 42 anos (DP=9,78; mín=29; máx=62). A maioria se considerava da cor branca (45,5%), majoritariamente casada ou em união estável (45,5%), com o catolicismo como religião predominante (68,2%). No que se refere à formação profissional, 63,6% dos entrevistados relataram ter entre sete e 20 anos de atuação profissional, e possuíam, além da especialização em enfermagem forense, formação *stricto sensu* (59,1%). O perfil de formação desses profissionais é apresentado na Tabela 1.

**Tabela 1:** Perfil da formação em enfermagem forense, Brasil, 2023

Variáveis		n	%
**Ano da formação em enfermagem forense**	2015	1	4,5
	2018	5	22,8
	2019	7	31,8
	2021	3	13,6
	2022	5	22,8
	2023	1	4,5
	Total	22	100
**Como adquiriu o título de especialista em enfermagem forense**	Especialização presencial	9	40,9
	Educação a Distância	2	9,1
	Prova de títulos das entidades de classe representativas da área	11	50
	Total	22	100
**Instituição que conferiu o título de enfermeiro forense**	Associação Brasileira de Enfermagem Forense	4	18,2
	Sociedade Brasileira de Enfermagem Forense	7	31,8
	Faculdade IDE - Universidade Cristo Redentor	3	13,6
	Outros	8	36,4
	Total	22	100

Entre os entrevistados do estudo, 12 (54,5%) possuem registro de especialista em enfermagem forense no Conselho Regional de Enfermagem (COREN) ao qual estavam vinculados. Quanto à vinculação às entidades representativas da área, 11 (50%) estavam associados à Sociedade Brasileira de Enfermagem Forense (SOBEF), seis (27,3%), à Associação Brasileira de Enfermagem Forense (ABEFORENSE), e cinco (22,7%) não estavam vinculados aos órgãos representativos da área.

Os entrevistados referem atuar no ensino e pesquisa (n=12), na assistência (n=10), na gestão (n=8) e no empreendedorismo (n=1), e alguns deles atuam em mais de uma área simultaneamente. Possuem outras especialidades assistenciais, administrativas e educacionais da enfermagem que estão relacionadas à: saúde pública; saúde mental; especialidades clínicas nos diferentes estágios da vida (neonatologia, pediatria, ginecologia e obstetrícia, gerontologia); urgência, emergência e terapia intensiva; gestão em saúde; administração e auditória hospitalar e em enfermagem; enfermagem do trabalho; educação profissional e bioética; entre outras. Os profissionais aplicam os conhecimentos forenses nas diversas áreas de atuação da enfermagem.

A partir da análise das entrevistas, emergiram três categorias temáticas: Disseminação e fortalecimento do ensino da enfermagem forense na graduação e pós-graduação com avanço dos estudos relacionados à área; Envolvimento político para o desenvolvimento de normativas nacionais que viabilizem a prática da enfermagem forense nos serviços relacionados; Vagas para enfermagem forense e sua inserção nos serviços especializados de identificação e combate da violência e nas emergências das unidades hospitalares.

### Disseminação e fortalecimento do ensino da enfermagem forense na graduação e pós-graduação com avanço dos estudos relacionados à área

Na primeira categoria temática, os entrevistados destacam a necessidade urgente de incluir a enfermagem forense nos currículos de graduação e pós-graduação como uma forma de garantir que os enfermeiros, desde a formação inicial, possuam conhecimentos básicos sobre o manejo de casos forenses, como acolhimento de vítimas de violência e condução dos encaminhamentos adequados. Como destacado por um dos entrevistados, um conhecimento mínimo sobre a atuação forense poderia proporcionar uma base sólida para que o profissional de enfermagem, mesmo sem ser especializado, possa atuar de forma adequada em situações de violência.

*É importante mudar toda essa dinâmica curricular das universidades. Os professores têm que estar atentos na área que ele trabalha e se atualizar quanto aos temas transversais relacionados à enfermagem forense e o que está sendo falado. Não precisa ser forense pra você ter o conhecimento básico que o enfermeiro generalista precisa ter acerca do dever dele perante o código de ética, encaminhamento, notificação, quando necessário, realizar denúncia. Não precisa ser forense pra fazer tal ação. O profissional, tendo na formação dele pelo menos uma noção geral de acolhimento e direcionamentos aos casos relacionados às questões forenses, já vai estar ajudando e vai estar respaldado pelo conhecimento básico adquirido na formação, na universidade.* (EF03)

*A gente não pode trabalhar algo se as pessoas não conhecem. A inserção dos conhecimentos da enfermagem forense a partir da graduação é fundamental. Com o contato na sua formação inicial e original, você vai poder despertar, mesmo que você não seja um enfermeiro forense posteriormente, mas você vai poder despertar pra alguns cuidados relacionados que você precisa ter. Eu defendo a inserção do conhecimento da enfermagem forense dentro da graduação.* (EF11)

*Instituir a enfermagem forense na grade curricular das universidades, porque o profissional enfermeiro, quando ele se profissionaliza e sai com o título pra atuar, ele sai muito despreparado, e ele pode inclusive ser prejudicado judicialmente às vezes* [...] *eu sei que é uma coisa difícil, que as faculdades não querem, até acham lindo, bem legal, mas na grade curricular, não, dificilmente como conteúdo extracurricular.* (EF08)

*Eu digo sempre nas minhas palestras: a gente precisa sensibilizar as instituições, seja ela pública ou privada, para que a enfermagem forense esteja de forma transversal em todos os conteúdos disciplinares que abrange a violência ou ela esteja como enfermagem forense enquanto um conteúdo com as nossas competências, para que a gente possa sensibilizar os novos aprendizes e os nossos egressos tenham a noção do que é a especialidade. Por outro lado, nós precisamos solidificar os programas de lato sensu, deixando-os com um conteúdo robusto, feito por pessoas experientes, que tragam de fato e de direito a formação que aquele profissional precisa para não invadir áreas dos outros e não deixar de fazer a sua área. Quando eu digo a área do outro, é área do outro profissional, de outra especialidade, a área do próprio enfermeiro.* (EF18)

Evidenciou-se a necessidade da ampliação do ensino da enfermagem forense desde a graduação até a pós-graduação *stricto sensu* por meio de estudos científicos robustos e com rigor metodológico, para que, assim, a enfermagem forense se fortaleça com a ampliação do conhecimento científico.

*O ponto de partida é a formação* [...] *ampliar a quantidade e a qualidade também, porque, quando você amplia a quantidade, você corre o risco da qualidade, de fazer uma pós-graduação totalmente teórica, sem nenhuma vivência de nenhuma área, nem de vestígio, nem de violência, nem de prisão, nem de desastre, e ficar com aquele título papel, que você não tem prática nenhuma, como eu conheço enfermeiros que são especialistas em UTI e não sabem o que é uma bomba de infusão. É sobre isso, para além da quantidade, qualificar, capacitar.* (EF13)

*A questão das pós-graduações, a tendência é começar a surgir outras. Quanto mais profissionais especialistas, maior será a visibilidade da área. Não existe um programa específico de enfermagem forense* [s*tricto sensu*]. *Tem que ser do interesse dos programas de pós-graduação de formar profissionais bem capacitados para estarem direcionando os estudos específicos da área e formando outros profissionais com excelência. Eu penso que é uma das questões que podem ter avanço com o desenvolvimento de pesquisas.* (EF03)

*Eu encaro que a gente pode contribuir muito em vários aspectos da enfermagem forense no Brasil e que não tem como não linkar várias das dificuldades sociais à nossa atuação e não ter pesquisa. O problema é que a gente precisa de apoio, recursos pra isso. A gente precisa avançar muito em pesquisa. Eu acho que as pesquisas em enfermagem forense são muito fracas comparada com os outros países. Precisa avançar nos estudos, nos tipos de estudo que nós fazemos. Uma das melhores maneiras da gente mostrar a nossa importância é mostrando pesquisa, estudos mais robustos, com um grande número de população para que as autoridades governamentais vejam a importância e veja o quanto que o serviço está ruim.* (EF09)

[...] *assim como a gente se espelha na enfermagem forense americana, que a gente possa levar a nossa prática daqui pra lá também, não só pra lá, mas pra outros países em desenvolvimento* [...] *a gente precisa escrever mais, precisamos ser mais científicos.* (EF15)

[...] *é um caminho que a gente precisa fazer esse enfrentamento para criar, além de uma área de uma linha de pesquisa, se criar um mestrado, um doutorado específico para o enfermeiro forense, porque, na hora que a gente tiver um mestrado específico, e não somente uma linha de pesquisa, eu tenho certeza que a gente vai ter muita produção científica, e eu acredito que a produção científica, ela é inquestionável. Que sejam produções científicas robustas, com pesquisas de campo, multicêntricas, e não só revisão de literatura, como a gente tem visto* [...] *são as universidades que precisam fazer esse enfrentamento. Enquanto a gente não tiver esse apoio das universidades, principalmente as públicas, a gente vai demorar um pouco para chegar lá, a gente vai chegar, ninguém vai ficar parado no meio do caminho, mas eu digo a universidade, pois ela traz o saber junto com ela; ela traz a pesquisa; ela traz a crítica; ela se autocrítica até quando vai fazer um trabalho de pesquisa: “não é esse caminho, não, esse caminho não serve para você, não, vamos fazer o outro”, e aí a gente vai fazendo as costuras para poder chegar.* (EF18)

### Envolvimento político para o desenvolvimento de normativas nacionais que viabilizem a prática da enfermagem forense nos serviços relacionados

Na segunda categoria temática, os entrevistados destacaram a importância de um movimento político coordenado que envolvesse não apenas os profissionais de enfermagem, mas também o COFEN, parlamentares e gestores públicos, para a criação de leis que assegurem a função do enfermeiro forense em concursos públicos e em sistemas de polícia científica, Institutos de Medicina Legal (IMLs) e outros serviços que atendem vítimas de violência.

*Sem o movimento político daqueles que estão se formando, nada acontecerá, porque é só com o movimento político que as coisas acontecem. Eu costumo dizer que qualquer ação administrativa, antes de tudo, ela é política. Os POPs, antes de serem administrativos, eles são políticos* [...]. (EF02)

*Eu vejo que deu um “boom”, principalmente nos últimos dois anos avançou bastante. Para avançar um pouco mais, precisa dos campos de atuação. Que a gente consiga conversar politicamente sobre isso com deputados, senadores, vereadores, estas pessoas que podem ajudar, que têm o poder político, que podem colocar também a especialidade no campo de trabalho, para que a gente saia um pouco do campo teórico e comece a ver na prática como isso funciona aqui no Brasil. Acredito que o avanço agora vai ser progressivo, só pra frente. Não tem como barrar a enfermagem forense no Brasil, a gente vai virar referência na América Latina, eu não vejo outros países latino-americanos se movimentarem como a gente tem se movimentado aqui para a especialidade avançar.* (EF04)

[...] *nós já avançamos, hoje já temos o CBO, tem a especialidade. Para avançar mais, precisamos de uma política mais agressiva, inclusive por parte do COFEN. Pressionar o legislativo, pressionar líderes para realmente se criar a figura do enfermeiro forense em concursos públicos, que seja para desenvolver perícia, que se possa usar no sistema penitenciário. Para a gente avançar mesmo, nós precisamos dessa atuação política e de uma política mais agressiva por parte do Conselho Federal* [...] *senão nós vamos ficar só em sonhos, nos projetos.* (EF05)

Exposta a necessidade desta articulação política como estratégia fundamental para o fortalecimento da área, destaca-se o papel do COFEN na criação de comissões estaduais como uma ação mobilizadora. Observa-se uma preocupação com a ausência de políticas públicas específicas e de recursos adequados para inserção dos enfermeiros forenses nas instituições, o que dificulta o aproveitamento desses profissionais. Além disso, os participantes reforçam a capilaridade da enfermagem no território nacional, defendendo a inclusão da enfermagem forense nas políticas de segurança pública, em articulação com os Ministérios da Saúde e da Defesa, como forma de ampliar o enfrentamento da violência e qualificar o atendimento às vítimas.

*A questão política vai ser o nosso grande apoio, porque, atualmente, o COFEN começou a formar comissões em vários estados* [...]. (EF10)

*Falta políticas públicas no sentido de ter recursos pra poder absorver os enfermeiros forenses e as instituições se apropriarem desses profissionais que estão se formando.* (EF09)

*A enfermagem é uma ciência capaz; possui profissionais realmente qualificados. Estamos em todos os lugares do país, a gente não encontra fórum em todos os municípios, mas encontramos enfermeiros em todos os municípios. A gente está de ponta a ponta. A enfermagem forense precisa ser inserida nas políticas de segurança pública, fazendo a junção aí com o Ministério da Saúde, Ministério de Defesa e suas secretarias estaduais e municipais no combate à violência.* (EF10)

### Vagas para enfermagem forense e sua inserção nos serviços especializados de identificação e combate da violência e nas emergências das unidades hospitalares

Na terceira categoria temática deste estudo, os profissionais apresentam a necessidade da realização de concurso público com vagas específicas para a área, assim como algumas possibilidades de inserção desse profissional para prestar assistência às pessoas que passaram por alguma situação de violência, não apenas como um serviço complementar.

*Um dos maiores ganhos será quando você pegar um edital de uma polícia científica e vê nas áreas interdisciplinares a enfermagem forense inserida lá ou dentro de um hospital um serviço parceiro do Instituto Médico Legal ou do Serviço de Verificação de Óbito, parcerias com o judiciário, com a delegacia. Então, tendo essas parcerias, vai começar a ter uma credibilidade e uma visibilidade maior* [...] *a questão do concurso públicos é um dos maiores desejos das próprias sociedades* [SOBEF, ABEFORENSE] *em ter os concursos voltados a essa área, porque a gente vê toda essa questão catastrófica acontecendo no país do aumento de violência. Eu espero que futuramente isso aí possa ter uma visibilidade maior.* (EF03)

*Na próxima década, eu enxergo mais enfermeiros forenses formados, registrados nos seus conselhos, para que, quando se puxar uma lista, tenha lá pelo menos 20 num estado, dez no outro, 15 no outro; se possa abrir um concurso a nível nacional pra enfermeiro forense; possa ter concursos dentro da saúde em si, os estados abrindo concurso vaga para enfermeiro em UTI, enfermeiro de emergência, e em enfermagem forense. Seleções simplificadas para enfermeiro forense, principalmente nas instituições que são referência pro atendimento de pessoas vítimas de violência, para a tão sonhada carreira dentro dos institutos de criminalística, dentro da perícia, da polícia científica, dos concursos policiais, dos tribunais. É muito a se trabalhar pra que isso realmente aconteça, hoje a gente almeja isso, a gente sonha com isso. Quem sabe isso possa ocorrer na próxima década. Ainda são portas que a gente vai bater muito, mas que vão se abrir. Precisamos ocupar esses espaços que existem. As pessoas só não sabem dos benefícios para sociedade que será ocupar esses espaços.* (EF07)

[...] *enquanto a gente não tiver concursos públicos para atuação do enfermeiro forense, a gente não vai ter muitas saídas, a gente não vai ter muito sucesso com essa área. Eu estudo porque eu gosto, é uma área que me dá prazer, é estranho falar de violência, de morte, de desastres falando de prazer, mas é uma coisa que me dá satisfação em ajudar quem mais precisa, mas eu sei que financeiramente, muito dificilmente, eu vou ter algum retorno com essa área.* (EF16)

Os resultados da pesquisa evidenciam um conjunto de expectativas e propostas em torno da efetiva inserção do enfermeiro forense nas estruturas de saúde e segurança pública. Os participantes ressaltam a necessidade de integrar esses profissionais às Secretarias Municipais e Estaduais de Saúde, promovendo o matriciamento das equipes da atenção primária, urgência e emergência, especialmente no atendimento às vítimas de violência em situações agudas. Destaca-se o desejo de que a enfermagem forense se torne referência nos serviços especializados de enfrentamento da violência sexual contra mulheres, crianças, idosos e populações privadas de liberdade. Os entrevistados também enfatizam o papel transformador que a atuação forense pode exercer dentro dos serviços, inspirando outros estados a adotar iniciativas semelhantes e contribuindo para qualificar o cuidado e acesso à justiça das vítimas.

*A inserção do enfermeiro forense nas Secretarias Municipais e Estaduais de Saúde fazendo um matriciamento das equipes de atenção primária, para as equipes de urgência e emergência. Que a gente tenha o enfermeiro forense no atendimento às vítimas de violência nas situações agudas, que são no serviço de urgência e emergência, com sala especializada. Que a gente realmente consiga ser referência no serviço de atendimento à violência sexual contra a mulher, criança, idoso no sistema penitenciário. Que a gente consiga a criação do cargo, principalmente nos IMLs. Talvez essa parte seja a mais complexa e a mais demorada, que é ter um concurso público com cargo específico pra gente. Mas a inserção nas secretarias, no programa de saúde escolar, e nas unidades de urgência e emergência, seja um caminho.* (EF14)

*Quero ver todos os concursos de polícia civil com o cargo de enfermagem forense, essas parcerias das redes, crescer mais e mais, ser algo que esteja dentro dos hospitais, por exemplo, aqui no hospital, gostaria muito de ter a vaga de enfermeiro forense disposta à urgência e emergência* [...] *tem alguns enfermeiros que atendem as vítimas de violência e às vezes nem sabem o que fazer, como abordar, encaminhar. Eu tenho essas expectativas.* (EF17)

*Olha, eu estava dando uma olhada na questão do concurso de Pernambuco, que o COREN de lá pediu para ser incluído a enfermagem forense como perito, eu achei fantástico. Eu acho que é isso, trabalho de formiguinha. Se um estado conseguir aprovar isso, não é nada para outro estado conseguir. Então, a partir do momento que o enfermeiro forense começar a fazer diferença na polícia científica, isso será um espelho, um exemplo bom para que os outros estados possam incluir a nossa especialidade, com nossa função, realizando um trabalho social muito bonito, muito bem feito, porque é isso, é para isso que a gente realmente fez a especialidade: para fazer a diferença.* (EF19)

## DISCUSSÃO

A enfermagem forense no Brasil é uma área emergente que apresenta desafios significativos tanto no que diz respeito à sua formação acadêmica, e ao reconhecimento político e legislativo quanto à inserção dos enfermeiros forenses em serviços especializados.

A formação acadêmica da enfermagem forense é um dos pilares para o fortalecimento dessa especialidade no Brasil. Estudo anterior destaca a importância de integrar esse conhecimento de forma transversal nos currículos universitários^(2,11)^. A enfermagem forense não se restringe à prática específica do perito, mas abrange competências essenciais para o manejo de vítimas de violência, a notificação de crimes e o acolhimento de situações de trauma^(12)^.

A inclusão de conteúdos forenses na formação básica de enfermagem, como o código de ética, as notificações obrigatórias e o acolhimento às vítimas de violência, é fundamental para garantir que todos os profissionais, mesmo os não especializados, saibam como proceder em situações críticas. Embora os cursos de graduação abordem temas relacionados à saúde e cuidados clínicos, a enfermagem forense ainda é frequentemente tratada como um tópico marginal ou, quando abordado, em módulos extracurriculares^(2,11)^. Essa realidade reflete uma lacuna significativa na formação dos enfermeiros brasileiros, exigindo uma mudança no currículo das universidades para que os enfermeiros generalistas também recebam uma preparação mínima, mesmo que não se especializem na área.

A qualificação das pós-graduações, com um foco mais prático e menos teórico, também foi um ponto discutido pelos entrevistados. O crescimento da quantidade de especialistas é importante, mas a qualidade da formação é ainda mais essencial, para evitar a criação de profissionais que, apesar de possuírem a titulação de especialista, careçam da vivência prática necessária para atuar na área^(13,14)^. É imprescindível que a formação especializada inclua experiências reais em cenários de violência, desastres e investigações, de forma a preparar o enfermeiro para os desafios e complexidades que surgem nessas situações^(15,16)^.

Além disso, a integração entre teoria e prática nas pós-graduações é fundamental para garantir que o enfermeiro não apenas entenda os conceitos jurídicos e forenses, mas que também tenha a habilidade de identificar vestígios e compreender as particularidades do atendimento às vítimas de violência^(17,18)^. Isso reforça a necessidade de mais estudos robustos que forneçam uma base científica sólida para a enfermagem forense, especialmente no Brasil, onde os investimentos em pesquisas científicas nesta área ainda são escassos em comparação a outros países^(19)^.

Nos Estados Unidos, a enfermagem forense é uma área consolidada tanto na formação acadêmica quanto na prática clínica. Nesse país, os cursos de graduação e pós-graduação em enfermagem forense são bem estabelecidos, com programas dedicados que incluem tópicos sobre a coleta de evidências, a documentação de lesões, a atuação judicial e a saúde da vítima. Além disso, muitos estados exigem que os enfermeiros forenses passem por certificações adicionais para trabalhar com vítimas de violência sexual, em um modelo de prática integradora entre o sistema de saúde e a justiça. Isso ajuda a formar profissionais mais bem preparados para atuar na interseção entre a enfermagem, a saúde pública e o direito^(17,18,20)^.

Esse modelo poderia ser um ponto de referência para o Brasil, onde a integração da enfermagem forense nos currículos universitários ainda é limitada. Como sugerido pelos entrevistados, a formação inicial poderia ser mais robusta, com um foco maior nas competências forenses, e poderia ser complementada por programas de pós-graduação que ofereçam uma especialização formal^(14)^.

Outro ponto essencial para o avanço da enfermagem forense no Brasil diz respeito ao envolvimento político dos profissionais da área. A criação de normativas nacionais que estabeleçam a atuação do enfermeiro forense nos serviços de saúde, como hospitais e unidades de emergência, bem como nas áreas da segurança pública e da justiça, é fundamental para garantir a inserção desse profissional no mercado de trabalho.

A atuação do enfermeiro forense é essencial não apenas para o cuidado da vítima, mas também para garantir a validação dos laudos médicos, coletar evidências que possam ser utilizadas em investigações criminais e fornecer testemunhos em tribunais^(21)^. Nesse sentido, a atuação política e a pressão das entidades, neste caso, representativas da enfermagem forense no Brasil, como o COFEN, a SOBEF e a ABFORNSE, são essenciais para garantir que o enfermeiro forense seja incluído como uma especialidade reconhecida e tenha seu papel institucionalizado em diferentes áreas, como saúde pública, segurança e justiça^(22)^.

A ausência de uma política pública sólida que incentive a contratação de enfermeiros forenses é uma barreira que impede a expansão dessa especialidade no Brasil. A falta de recursos financeiros e a ausência de concursos públicos com vagas específicas para a área dificultam o acesso desses profissionais aos espaços especializados. Como apontado por um dos entrevistados, a pressão política deve se intensificar para que a enfermagem forense se torne parte da política de saúde pública, especialmente em áreas de combate à violência, como a violência sexual, violência doméstica e crimes contra crianças e idosos^(23-26)^.

Nos Estados Unidos e no Reino Unido, os enfermeiros forenses possuem um respaldo legal mais forte, com regulamentações específicas para a profissão e um papel claro dentro do sistema de justiça. O sistema de saúde britânico, por exemplo, tem investido em programas de treinamento contínuo para enfermeiros forenses, criando políticas públicas de inclusão da enfermagem forense no atendimento às vítimas de violência sexual, doméstica e outras formas de abuso. No Reino Unido, os enfermeiros forenses têm o papel de coletar provas, realizar entrevistas forenses e até atuar como testemunhas em tribunal. Essa integração da enfermagem no sistema judicial é protegida por uma série de regulamentações, o que garante a qualidade do atendimento e a segurança jurídica dos profissionais e das vítimas^(27-29)^.

No Brasil, a inexistência de políticas públicas claras e a falta de apoio político para criação de concursos específicos para enfermeiros forenses são vistos como obstáculos significativos para expansão da prática da enfermagem forense. Contudo, a crescente mobilização de entidades como o COFEN e a inclusão da enfermagem forense nas pautas de algumas esferas políticas podem ser os primeiros passos para institucionalização dessa prática no sistema de saúde e justiça nacional.

A inserção de vagas específicas para enfermeiros forenses, especialmente em serviços como IMLs, hospitais de referência, delegacias e outras instituições de segurança e justiça, foi uma demanda recorrente nas entrevistas. Muitos entrevistados expressaram o desejo de ver a enfermagem forense sendo integrada aos concursos públicos, com vagas específicas para a área.

A criação de vagas para enfermeiros forenses em instituições públicas e no sistema penitenciário poderia proporcionar uma credibilidade maior ao trabalho desses profissionais e também garantir que as vítimas de violência sejam atendidas de maneira mais eficiente e qualificada^(30-32)^. O enfermeiro forense tem a habilidade de coletar evidências forenses e prestar cuidados médicos de emergência enquanto assegura a preservação da cadeia de custódia das provas. Esse profissional, portanto, seria um elemento essencial nas investigações criminais e no processo judicial, desempenhando um papel tanto na saúde quanto na justiça^(29,33-35)^.

A inserção desses profissionais em unidades de emergência e no atendimento às vítimas de violência sexual e violência doméstica é vista como uma forma de melhorar a qualidade do atendimento, proporcionando apoio técnico à equipe médica e policial^(27,36)^. No entanto, como apontado pelos entrevistados, a falta de concursos públicos com vagas específicas e de infraestrutura nos hospitais e IMLs é obstáculo significativo para inserção dos enfermeiros forenses nessas instituições.

Experiências internacionais, como na Austrália, podem servir de exemplo para o Brasil nesse sentido. A inserção de enfermeiros forenses nas equipes de resposta a emergências, como na linha de frente do atendimento às vítimas de violência sexual e acidentes traumáticos, é uma prática consolidada no país. Além disso, enfermeiros forenses australianos frequentemente trabalham em colaboração com forças policiais e outros órgãos governamentais em investigações criminais, demonstrando a importância da interdisciplinaridade. A criação de vagas específicas para enfermeiros forenses em concursos públicos também é uma realidade na Austrália, onde o governo reconhece a necessidade de profissionais capacitados para atuar em situações de violência e tragédias^(22,37,38)^.

Na perspectiva projetiva para a próxima década, a enfermagem forense no Brasil tende a se consolidar como uma especialidade estratégica para a interface entre saúde, justiça e segurança pública. A expectativa é de que os avanços no ensino e pesquisa se traduzam em maior qualificação profissional, com a inserção definitiva da temática nos currículos de graduação e a ampliação de programas de pós-graduação *lato* e *stricto sensu* capazes de produzir conhecimento científico robusto e aplicável à realidade brasileira. Paralelamente, prevê-se o fortalecimento da mobilização política da categoria, com o engajamento de entidades representativas e órgãos reguladores na construção de normativas nacionais que assegurem a atuação do enfermeiro forense em diferentes esferas institucionais.

Esse movimento deverá favorecer a criação de concursos públicos e a abertura de vagas específicas para a especialidade em serviços de saúde, unidades hospitalares de urgência e emergência, IMLs, e delegacias especializadas, respondendo de forma mais efetiva às demandas crescentes de enfrentamento da violência. Assim, a próxima década se desenha como um período de expansão, institucionalização e reconhecimento social da enfermagem forense no Brasil, com potencial para impactar positivamente tanto a qualificação do cuidado prestado às vítimas quanto o fortalecimento das políticas públicas de segurança e justiça.

### Limitações do estudo

Uma das limitações deste estudo é metodológica, considerando que o autor principal não conseguiu realizar devolutiva das entrevistas para as respectivas conferências dos participantes. Outra limitação foi o número de participantes, especialmente por se trata de pesquisa de alcance nacional na maior área da saúde, que é a enfermagem. Contudo, o estudo evidencia a necessidade de expandir e divulgar ainda mais esse campo de conhecimento. Os resultados apresentados destacam como pontos positivos a descrição atual dos profissionais atuantes e comprometidos com uma área em crescimento na enfermagem, além de trazer algumas perspectivas concretas para a especialidade. A participação de profissionais dedicados potencializa a criação de estratégias para o desenvolvimento da área, além de fornecer subsídios para futuras iniciativas de aplicação da enfermagem forense nos serviços de saúde.

### Contribuições para as áreas da enfermagem, saúde e políticas públicas

A partir da perspectiva dos participantes, este estudo reforça a importância de o conhecimento em enfermagem forense estar presente da formação generalista à pós-graduação, possibilitando o avanço dos estudos e o fortalecimento da área, viabilizando o desenvolvimento de potencialidades/habilidades cognitivas dos estudantes e profissionais de enfermagem, uma vez que possibilita a compreensão dos fenômenos e as dimensões trabalhados nesta especialidade, bem como a sua responsabilidade profissional nesses contextos. O envolvimento político é fundamental para o desenvolvimento de normativas que permitam a prática do enfermeiro forense nos serviços especializados e relacionados à área. Esse fortalecimento poderá conduzir a implantação dos cargos de enfermeiro forense nos serviços públicos de saúde e seu espaço nas emergências hospitalares.

## CONSIDERAÇÕES FINAIS

A análise dos resultados evidenciou que a enfermagem forense no Brasil, embora ainda esteja em processo de consolidação, apresenta perspectivas claras de fortalecimento e expansão para a próxima década. Foi possível conhecer, a partir dos achados, que existe uma convergência de metas estratégicas entre os profissionais da área, organizadas em três eixos principais: a disseminação e qualificação do ensino da especialidade desde a graduação até a pós-graduação *stricto sensu*, com vistas à produção científica robusta e aplicável; o engajamento político-institucional como caminho essencial para criação de normativas nacionais que legitimem e regulamentem a prática do enfermeiro forense; e a inserção formal desses profissionais em concursos públicos e serviços especializados, especialmente no enfrentamento da violência em contextos hospitalares, IMLs e sistemas de segurança pública.

Tais metas convergem para construção de uma identidade profissional mais sólida, capaz de responder às demandas sociais emergentes relacionadas à violência e violação de direitos humanos, ao mesmo tempo em que posicionam a enfermagem forense como campo estratégico de interface entre saúde, justiça e segurança. Conclui-se, portanto, que os achados desta pesquisa projetam para a próxima década um cenário de maior institucionalização, reconhecimento e impacto social da enfermagem forense no Brasil, desde que acompanhados de investimentos em formação, pesquisa, políticas públicas e criação de espaços de atuação específicos que assegurem não apenas a visibilidade, mas também a efetividade da contribuição desta especialidade para a sociedade.

## Data Availability

Os dados de pesquisa estão disponíveis no corpo do artigo.
